# Antimicrobial resistance in fish and poultry: Public health implications for animal source food production in Nigeria, Egypt, and South Africa

**DOI:** 10.3389/frabi.2022.1043302

**Published:** 2022-11-10

**Authors:** Ekemini M. Okon, Reuben C. Okocha, Babatunde T. Adesina, Judith O. Ehigie, Olayinka O. Alabi, Adeniran M. Bolanle, N. Matekwe, Babatunde M. Falana, Adebisi M. Tiamiyu, Isaac O. Olatoye, Olufemi B. Adedeji

**Affiliations:** ^1^ Department of Animal Science, College of Agricultural Sciences, Landmark University, Omu-Aran, Nigeria; ^2^ Department of Animal Sciences and Aquatic Ecology, Faculty of Bioscience Engineering, Ghent University, Ghent, Belgium; ^3^ Landmark University SDG 13 (Climate Action Research Group), Omu-Aran, Nigeria; ^4^ Landmark University SDG 14 (Life Below Water Research Group), Omu-Aran, Nigeria; ^5^ ICBAS – School of Medicine and Biomedical Sciences, University of Porto, Porto, Portugal; ^6^ Faculty of Sciences, University of Porto, Porto, Portugal; ^7^ Department of Agriculture Environmental Affairs, Rural Development and Land Reform, Veterinary Services, Cape Town, South Africa; ^8^ Department of Biosciences and Biotechnology, University of Medical Sciences, Ondo City, Ondo State, Nigeria; ^9^ Department of Veterinary Public Health and Preventive Medicine University of Ibadan, Ibadan, Nigeria

**Keywords:** antimicrobial resistance, antibiotics, antimicrobials, public health, one health, systematic review

## Abstract

Antimicrobial resistance (AMR) is a significant threat to global public health. Specifically, excessive usage of antimicrobials in food animal production is one significant reason for AMR development in humans. Therefore, it is essential to identify the trends of AMR in fish and poultry and develop better surveillance strategies for the future. Despite this imperative need, such information is not well documented, especially in Africa. This study used a systematic review to assess AMR trend, spatial distribution, and incidence in fish and poultry research in Nigeria, Egypt, and South Africa. A literature assessment was conducted for published studies on AMR between 1989 and 2021 using the Scopus and Web of Science databases. One hundred and seventy-three relevant articles were obtained from the database search. Egypt was the leading exponent of antimicrobial resistance research (43.35%, 75 studies), followed by Nigeria (39.31%, 68 studies), then South Africa (17.34%, 30 studies). The majority of the antimicrobial resistance studies were on poultry in Egypt (81%, 61 studies), Nigeria (87%, 59 studies), and South Africa (80%, 24 studies). Studies on fish were 17% (13 studies), 9% (6 studies), and 10% (3 studies) in Egypt, Nigeria, and South Africa, respectively. Antimicrobial resistance patterns showed multiple drug resistance and variations in resistant genes. AMR research focused on sulfamethoxazole groups, chloramphenicol, trimethoprim, tetracycline, erythromycin, and ampicillin. Most studies employed the disk diffusion method for antimicrobial susceptibility tests. Among the four mechanisms of AMR, limiting drug uptake was the most reported in this study (both in fish and poultry). The findings reveal public and environmental health threats and suggest that it would be useful to promote and advance AMR research, particularly for countries on the global hotspot for antimicrobial use.

## 1 Introduction

Aquaculture through fish production can provide a cheap supply of protein as the human population continues to increase globally (Colgrave et al., 2021). The need to meet the growing animal-source protein demand by the teeming global population has culminated in global aquaculture expansion. This expansion could be matched by the increased use of medicated feeds which are cheap and easily accessible for widespread use across farms to prevent bacterial diseases, especially in developing countries (Ibrahim et al., 2020). Due to their crucial importance to human medicine, in-feed usage of antimicrobials is usually deemed inappropriate for use in animal production. Thus, utilising a diverse range of these antimicrobial agents, including those used in human medicine, guarantees their persistence in the aquatic environment for an extended period. However, over an extended period, the persistence in the aquatic environment could lead to the development of antimicrobial resistance (Bilal et al., 2020).

Besides fish production, poultry contributes considerably to human nutrition and ensures food security by providing humans with protein, energy, and vital micronutrients (Mottet and Tempio, 2017). Poultry is one of the world’s most widely consumed meats. Large doses of antimicrobials are often used across farms to prevent and treat diseases and increase poultry flocks’ growth. Globally, chicken meat is produced in almost 90 billion tonnes per year, according to FAOSTAT, 2017 data, making it the most widely farmed species. The low cost of production and lack of religious and cultural restrictions on its use are the key reasons for this (Heise et al., 2015; Gavahian et al., 2019).

In most countries, antimicrobials are used in fish and poultry production. They are usually administered orally to prevent and treat diseases and enhance growth and productivity (Agunos et al., 2012; Landoni and Albarellos, 2015). On the other hand, antimicrobial-resistant pathogens in poultry can result in treatment failure and financial losses. In addition, the pathogens can also cause bacteria resistant to antibiotics (including zoonotic pathogens) that are hazardous to humans (Helmi et al., 2020).

In recent years, a substantial number of studies have focused on this research area, indicating the relationship and contributions of antibiotic usage to AMR in animals (Bennani et al., 2020; Kasimanickam et al., 2021). Specifically, excessive usage of antimicrobials in food animal production is one significant reason for AMR development in humans (McLain et al., 2016). Although millions of human lives have benefited from antimicrobials, the majority (73%) of such antimicrobials are those utilised for animal production (Van Boeckel et al., 2017). The benefits (disease treatment, enhanced growth and productivity) derived account for the widespread and increasing use of animal antimicrobials in production.

Many factors, such as growth promotion and disease, can influence the need for chemotherapeutic intervention in animal production. In addition, factors such as the therapeutic use of antimicrobials on the farm, agricultural runoff, and human pollution contribute to AMR in animals. In fish production, for instance, the most prevalent way to administer antimicrobial drugs is through broadcasted medicated feeds. The most extensive and potent fish ingest most of the medicated feed, ensuring a required medical outcome of the drug administered. However, the weaker, sick, and/or smaller fish will likely have limited access to it and become underdosed, making them far more susceptible to disease and death (Coyne et al., 2004; Austin and Austin, 2016).

It is widely known that antimicrobial exposure aids the development of drug resistance in fish and aquatic pathogens. In fish production, particularly intensive farming, antibiotics may be used indiscriminately. Indiscriminate antimicrobial use could result in bacterial resistance and the accumulation of antibiotic residues in aquaculture products such as fish (Chen et al., 2020). Also, a fundamental association between the usage of certain antimicrobials and an increase in Antimicrobial Resistant Bacteria (AMRB) has been demonstrated in various studies (Tendencia and de la Peña, 2001; Tsai et al., 2010). Antimicrobial-resistant pathogens released into the environment from agricultural runoff or fish farms can impact the general distribution of AMR *via* microbial communities. In a pond environment, integrated farming greatly favoured the development of antimicrobial-resistant microorganisms (Petersen et al., 2002). The leaching of antimicrobial compounds may also enhance the spread of antibiotic-resistant animal and zoonotic pathogens during fish production into nearby aquatic environments.

There is a direct relationship between antibiotics and the emergence of AMR in fish and human infections. Studies have highlighted that bacteria may convey resistance genes to the next generation (Miller and Harbottle, 2018). Another research suggests that the development of AMR in aquaculture environments may lead to AMR in bacteria or human diseases. This finding highlights the possibility of a relationship between antibiotic usage and potential effects on human health (Furushita et al., 2003; Lekshmi et al., 2017).

In aquaculture, Gauthier (2015) indicated that fish are reservoirs of zoonotic disease, infecting both the host and humans through food-borne diseases or direct contact at the aquaculture facility. In addition, other bacteria could infect fish handlers during the production process, including *Aeromonas hydrophilia*, *Photobacterium damselae*, *Vibrio vulnificus*, *Streptococcus iniae*, and *Mycobacterium marinum* (Haenen et al., 2013; Ogbonna and Inana, 2018). Similarly, food-borne diseases are primarily caused by *Clostridium* spp., *Listeria monocytogenes* and *Aeromonas* spp. (Cortés-Sánchez et al., 2019; Noor, 2019), and resistance to these bacteria could negatively impact human health through infections. Besides infections, such bacteria can produce AMR determinants and transmit them to other human diseases (Kerry et al., 1996; Tendencia and Peña, 2001). As a result, the emergence of AMR during fish production may contribute to the emergence of AMR in human infections (Furushita et al., 2003; Reverter et al., 2020).

Antimicrobial genetic determinants are found in bacteria from both aquatic and terrestrial habitats (Cabello et al., 2013). The clinical significance of antibiotic-resistance genes is determined by their prevalence, genomic context, and molecular mechanism. The mechanism varies between species and includes chromosomal mutations, inactivation, drug modification, and active efflux. Quinolones (such as enrofloxacin, oxolinic acid, and flumequine), phenicols (such as florfenicol), and tetracyclines (such as oxytetracycline) are among the most commonly used antibacterial drugs in fish production (Miranda et al., 2013). Quinolone resistance is typically acquired through chromosomal mutations in topoisomerase genes. Quinolone resistance can also be acquired through mutations, thus limiting drug accumulation by boosting efflux or decreasing absorption (Drlica and Zhao, 1997; Ruiz, 2003). Active efflux, drug modification, target mutation ribosomal protection, and tetracycline inactivation are all tetracycline resistance mechanisms (Nguyen et al., 2014). Specific and non-specialised drug transporters, specific hydrolases, and RNA methyltransferases are among the mechanisms of florfenicol resistance (Long et al., 2006; Tao et al., 2012; Li et al., 2020).

Egypt is the most productive aquaculture producer in Africa, followed by Nigeria. According to 2018 data, Egypt and Nigeria dominated African aquaculture production, with nearly 14 million metric tonnes and 263,000 metric tonnes, respectively (FAO, 2020; Kaleem and Sabi, 2020). With roughly one million tonnes produced yearly, Egypt today has the highest aquaculture production, particularly fish production in Africa (Shaalan et al., 2018). This rapid expansion is attributed to the sector’s transition from semi-intensive to intensive fish production systems (FAO, 2003-2021). Data shows that aquaculture, particularly fish production, has increased significantly in Nigeria. In 2000, production increased from 25,718 metric tonnes (MT) worth $56.6 million to 296,191 MT worth $848.6 million in 2017. In South Africa, the poultry industry has grown following global trends in most developed countries. The industry is dominated by a small number of completely integrated large commercial producers and several small-scale producers. Poultry products are the most widely produced, inexpensive, and consumed animal protein in the country, enhancing the aim of achieving its zero-hunger targets (SAPA, 2016). The industry is the most important part of their agricultural economy, accounting for over 16% of their gross domestic product. This development accounts for the choice of the three African countries for this study relative to an increased usage of antimicrobials.

AMR development in animals is majorly connected to the improper use of antimicrobial drugs. With these trends in fish and poultry, antimicrobials are likely to increase the incidence of devastating and lethal diseases (Michael et al., 2014). The use of antimicrobials in food animals such as fish and poultry has increased in recent years. Therefore, it is essential to identify the patterns of AMR in fish and poultry and develop better surveillance strategies for the future. This study used the Preferred Reporting Items for Systematic reviews and Meta-Analyses (PRISMA) to assess the trend, spatial distribution, and incidence of AMR in fish and poultry in Nigeria, Egypt, and South Africa.

This study is organised as follows: Section 2 presents the methods and approaches using the PRISMA procedures. Section 3 reports the results: the trend of publications, spatial distribution, the incidence of resistance genes, and characteristics of included studies. Finally, section 4 discusses the results, highlights future research directions, and concludes.

## 2 Methodology

### 2.1 Study design

This study used a mixed research method described by Robson and McCartan (2016). This method included an electronic literature search, literature appraisal, and secondary data analysis from published articles on antimicrobial resistance in Egypt, Nigeria, and South Africa.

### 2.2 Literature search methodology

An electronic literature search was carried out utilising the databases provided by Scopus and ISI Web of Science. The literature study was systematically conducted in reference to the Preferred Reporting Items for Systematic Reviews and Meta-Analyses (PRISMA) protocol recommendations (Moher et al., 2019). The PRISMA procedure was used to establish the present/comparative state of fish and poultry research concerning antibiotic resistance in Egypt, Nigeria, and South Africa.

The search range from 1989 to 2021 was chosen to capture all relevant articles. An analysis of studies on antimicrobial resistance was conducted with reference to the trend of published studies, distribution, the pathogen of interest, sample type, sample method, and mechanism of resistance. The captured articles were utilised in the data categorisation and analysis of pertinent research gathered throughout the investigation. Between January 1989 and December 2021, primary papers were obtained from the search databases using the search terms described in **Table 1**.

**Table 1:** Description of literature search terms using the Scopus and ISI Web of Science databases.

Items	Boolean keywords
Location	Egypt OR Nigeria OR South Africa
Discipline	Fish OR poultry OR health
Population	Antimicrobials OR antibiotics OR salmonella OR E. coli
Outcome	Identification OR residues OR investigation OR characterisation OR use OR resistance OR frequency OR occurrence OR incidence OR prevalence

**Figure 1:**
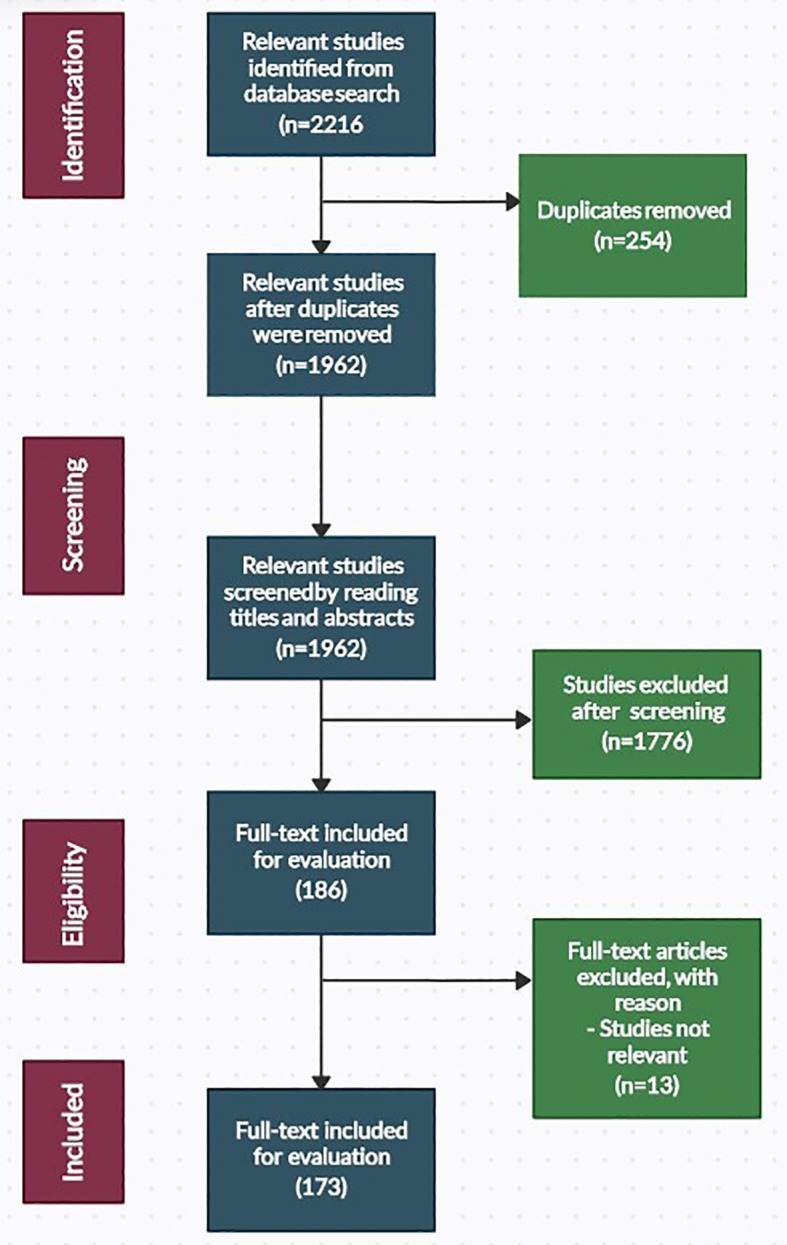
Overview of the PRISMA procedure used in this study.

The literature search was done in the “Topics” section of ISI Web of Science and the “Article Title, Abstract, and Keywords” section of the Scopus database. Both databases were set to capture articles published in the English language only. An initial search of the databases yielded 2216 articles, of which 173 were included in this study. A summary of the search procedure is presented in **Figure 1**.

### 2.3 Eligibility criteria

For a study to be eligible for inclusion, it was expected to relate to antimicrobial resistance with a focus on fish or poultry. No matter the research’s specifics, such studies could occur anywhere in Egypt, Nigeria, or South Africa. When considering the exclusion of a study, the primary criterion was whether it had “No concrete connection with antimicrobial resistance”. Another rationale for excluding a study was those conducted outside the study area. Studies published in other sources, such as editorials, conference abstracts, books, and book chapters, were also excluded. These types of studies are categorised here as “Others”.

### 2.4 Study selection

Study selection was conducted following the removal of duplicate reference items (254). The remaining 1962 reference items were then subjected to manual screening by reading the titles and abstracts or the complete texts. Following screening and full-text evaluation, 1789 reference items that did not meet the eligibility for inclusion were rejected. An outside expert handled any discrepancies regarding the study eligibility. Full-text copies of relevant articles that matched the criteria for inclusion (173 references) were evaluated.

### 2.5 Data collection and categorisation

Studies on antimicrobial resistance in the three countries were categorised into two research themes: fish and poultry. Studies relevant to the topic were extracted and categorised appropriately (category of study, sample type, sample method, and mechanism of resistance).

### 2.6 Data analysis

The data gathered in the three countries from the search databases were subjected to descriptive and quantitative analysis using Microsoft Excel software.

## 3 Results

This section presents the results of the trend of research publications, the spatial distribution of published studies, a comparison of published studies between fish and poultry among the countries, and an analysis of selected documents on antimicrobial resistance. The results below indicated that research in antimicrobial resistance varied among the countries with more focus on poultry.

### 3.1 Trend of publications on antimicrobial resistance in Nigeria, Egypt, and South Africa

**Figure 2:**
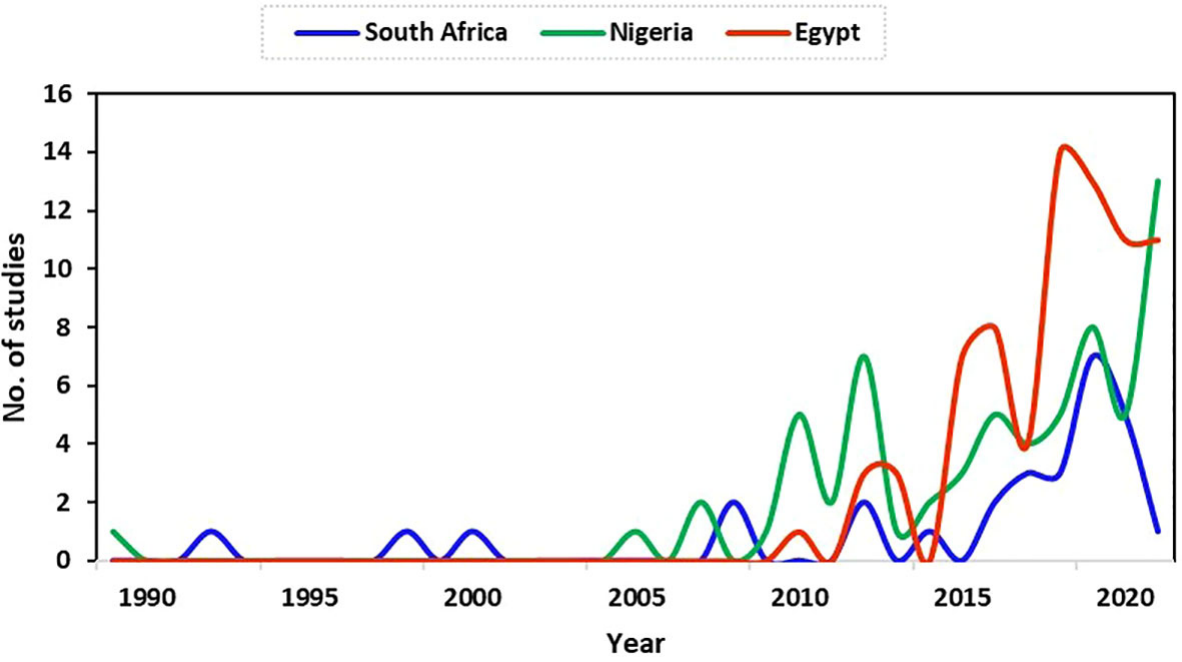
Trend of relevant publications on antimicrobial resistance in fish and poultry in Nigeria, Egypt, and South Africa.

The data and focus of this study are on antimicrobial resistance in fish and poultry. Thus, studies reported and analysed in this study focused on fish and poultry. There were limited studies on antimicrobial resistance for fish and poultry in Egypt during the early years until 2010 (1 study). From 2015 (7 studies), the number of studies increased to a peak in 2018 (14 studies). Between 2015 (7 studies) to the end of data collection for this study (September 2021), there were 68 studies (91%) in total in Egypt. The result shows a higher increase in the research field in recent years in Egypt compared to South Africa and Nigeria (**Figure 2**).

The first publication on antimicrobial resistance for fish and poultry during this study was in Nigeria in 1989. The study focused on the link between antibiotic usage and the development of drug resistance in *Escherichia coli* strains from poultry in a battery system. After that, no publication was obtained in some years within our research scope in Nigeria, as shown in **Figure 2**. In 2007, only two (2) studies on antimicrobial resistance were found, which stood as the highest number of publications until 2010. The number of publications increased from this period (although with fluctuations) to a peak of 13 studies in 2021 at the end of data collection for this study. This period marked more antimicrobial resistance research (specifically for fish and poultry) in Nigeria, as observed in this study.

Generally, the number of research publications on antimicrobial resistance in fish and poultry in South Africa increased from 1992 (1 study), with greater numbers found in later years from 2017 to a peak in the number of publications obtained in 2019 at the time of data collection for this study (**Figure 2**).

### 3.2 Spatial variation of antimicrobial resistance research publications in Nigeria, Egypt, and South Africa

**Figure 3:**
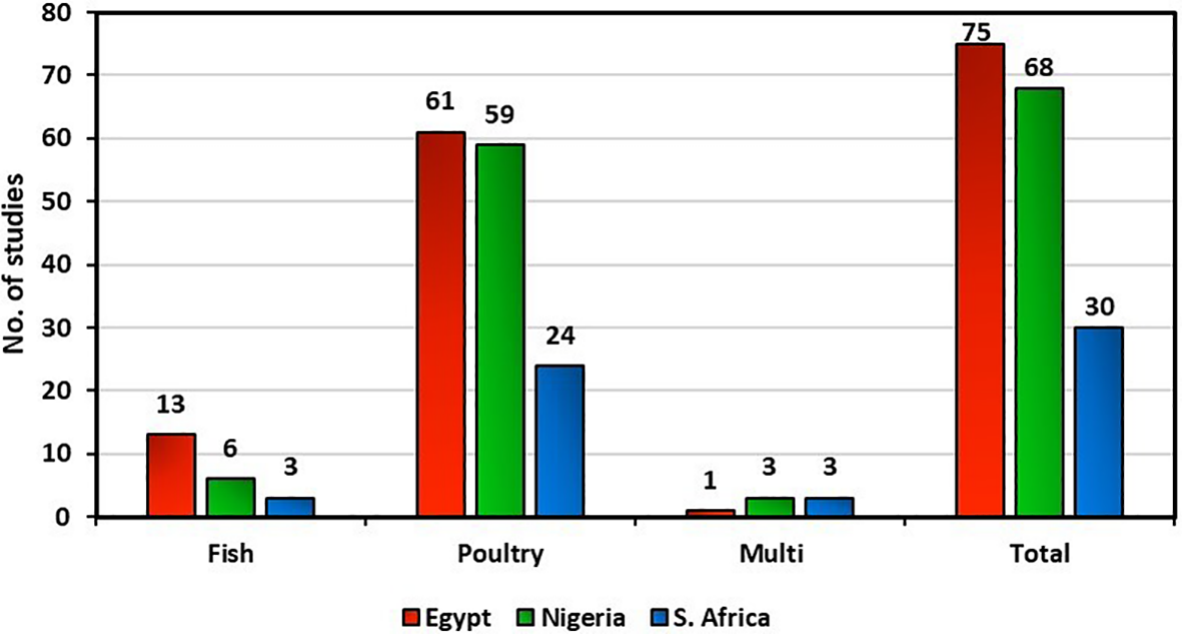
Comparison of published studies on antimicrobial resistance in fish and poultry in Egypt, Nigeria, and South Africa.

Studies on antimicrobial resistance in poultry were published more in Egypt, Nigeria, and South Africa than in fish. Studies on antimicrobial resistance in fish received comparatively little attention across the countries under study. Egypt was the leading exponent of antimicrobial resistance research (43.35%, 75 studies), followed by Nigeria (39.31%, 68 studies), then South Africa (17.34%, 30 studies). The majority of the antimicrobial resistance studies were on poultry in Egypt (81%, 61 studies), Nigeria (87%, 59 studies), and South Africa (80%, 24 studies). Studies on fish were 17% (13 studies), 9% (6 studies), and 10% (3 studies) in Egypt, Nigeria, and South Africa, respectively (**Figure 3**).

### 3.3 Incidence of resistance genes from various samples in fish and poultry research

**Figure 4:**
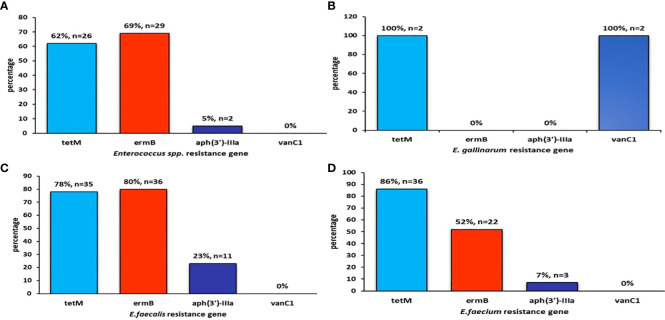
The figure shows the prevalence of **(A)** *Enterococcus spp* resistance gene, **(B)** *E. gallinarum* resistance gene, **(C)** *E. faecalis* resistance gene, **(D)** *E. faecium* resistance gene

**Figure 5:**
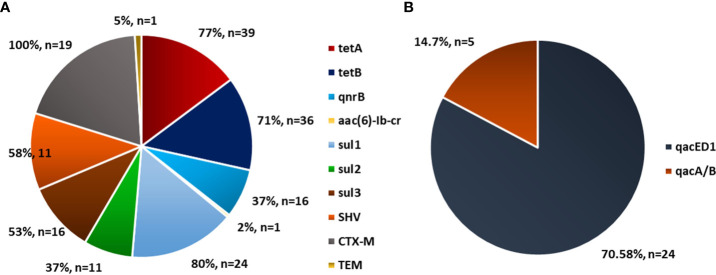
Prevalence of antibiotic resistance gene in *Escherichia coli* in poultry. Source: **(A)** Mclver et al. (2020), n=174 and **(B)** Ibrahim et al. (2019), n=34.

**Figure 6:**
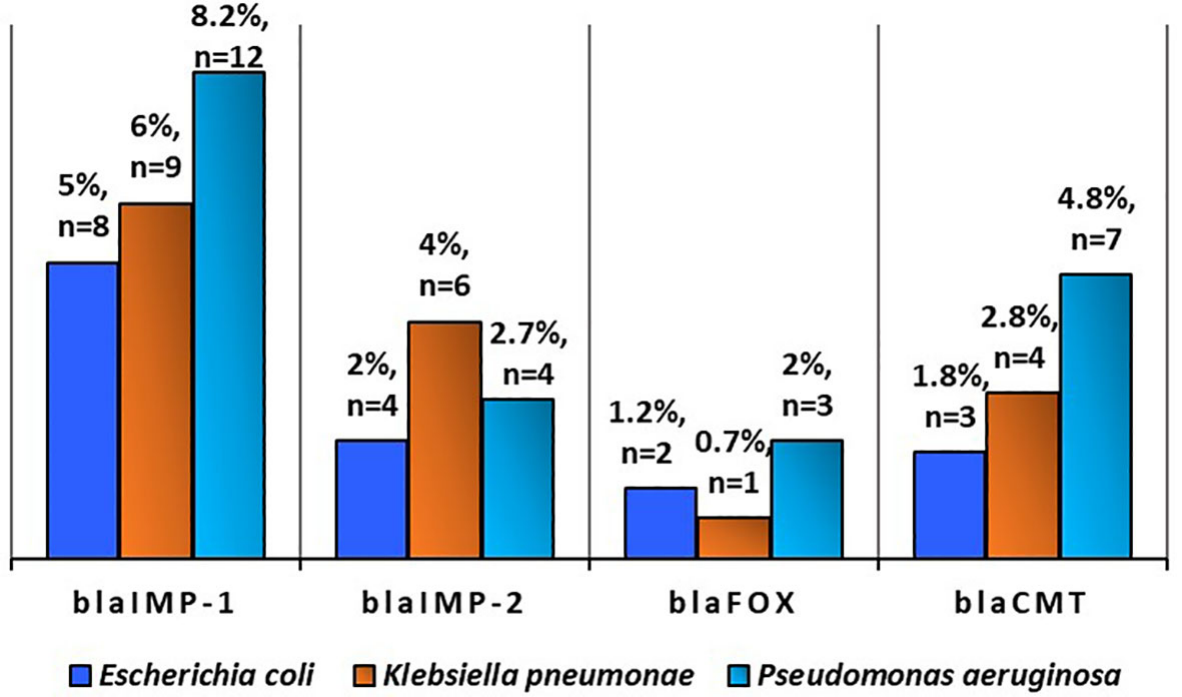
Prevalence of antibiotic resistance gene in *Escherichia coli* (n=168), Klebsiella pneumonae (n = 141), and Pseudomonas aeruginosa (n = 147) in poultry (Source: Ejikeugwu et al., 2021).

This study further explored the incidence of antimicrobial-resistance genes reported in poultry and fish research. Specific resistance genes reported in various studies are highlighted with reference to their resistance rate, expressed in percentages (**Figures 4**–**6**). One of the most well-known bacterial resistance mechanisms includes extended-spectrum β-lactamases (ESBL), which are mediated by plasmid genes (Ceccarelli et al., 2019). A significant contributor to resistance to expanded-spectrum β-lactam antibiotics is gram-negative organisms that produce ESBL (Castanheira et al., 2021). Lazarus et al. (2015) showed that the resistance mechanism could be achieved through the horizontal transfer of mobile genetic elements in both the extra-intestinal and intestinal environments. Furthermore, β-lactam antimicrobials like carbapenems, monobactams, penicillins, and cephalosporins can be hydrolyzed by ESBL (King et al., 2017). Thus, it becomes difficult to treat these infections (Bush and Fisher, 2011). Compared to non-ESBL producers, ESBL producers had a higher resistance to several different types of antibiotics (Teklu et al., 2019).

### 3.4 Characteristics of studies on antimicrobial resistance in fish and poultry

Further, the analysis of selected documents on antimicrobial resistance was conducted to understand the characteristics of some of the included publications. Methods for susceptibility tests across the thematic areas of AMR research in Egypt, Nigeria, and South Africa were majorly by the Kirby Bauer disc diffusion test. Also, limiting drug uptake was the central mechanism of antimicrobial resistance across the studies. A detailed analysis of these studies is presented in the **Supplementary Material** (**Tables 2**–**4**).

## 4 Discussion

### 4.1 Trend of publications on antimicrobial resistance in fish and poultry

The global expansion of fish and poultry production to support human food demand, no doubt, is concerned with some environmental risks (Kalantzi and Karakassis, 2006). One of the ways to tackle these risks is through appropriate research and communication of findings. However, research focus depends on various factors (awareness, funding, availability of relevant and skilled human resources, research interest, etc.).

Research findings are a significant source of key information for development and can provide a clearer perspective of a specific context of interest while mapping out future efforts and directions. Therefore, the findings of this study could be influenced by different factors. Among the factors are the researcher’s interest, motivation, funding availability, and conducive institutional platform for research (Ghabban et al., 2019). These factors have been reported to influence scholarly publishing. In addition, as Carayol and Matt (2004) noted, the number of research institution(s) and researchers in a particular field could influence research and publication outcomes.

This study showed limited studies on antimicrobial resistance for fish and poultry (hereafter AMRFP) during the early years. The result might be due to the limited use of antimicrobials during these periods. However, in subsequent years, AMR research began to gain more momentum, signifying increasing research interest in the field. In addition, the increasing human population with a corresponding increase in demand for animal-source proteins could have sparked the need for more antimicrobial use. Thus, reports on diverse adverse effects might have influenced the need for more research in the field compared to the preceding years.

Furthermore, there was an increase in fish and poultry research fields in recent years in Egypt compared to Nigeria and South Africa. Egypt is the largest fish producer in Africa, and the high production volume suggests that many veterinary antibiotics are utilised as therapeutic agents and growth promoters in production. Moreover, inappropriate use of antimicrobial drugs (Andersson, 2003) could be an essential contributor to the research intensification in AMR research due to the development of drug resistance.

In Nigeria and South Africa, more AMRFP research was also observed recently. The result might be connected to the increasing scientific discussion over the potential negative impacts of AMR, as reported in several studies (Jacobs and Chenia, 2007; Mamza et al., 2010; Igbinosa, 2016; Ajayi et al., 2019; Molechan et al., 2019; Mclver et al., 2020). Therefore, these studies must highlight recommendations for best-practice applications of antibiotics in food production, public health, and policy settings.

### 4.2 Spatial distribution of fish and poultry AMR publications

The present study shows that Egypt, Nigeria, and South Africa published more research on antibiotic resistance in poultry. Comparatively, little attention was paid to studies on antibiotic resistance in fish. Although aquaculture is a crucial sector in Egypt and Nigeria, AMR research in poultry received more focus than fish in these countries. Over the years, Egypt and Nigeria have been Africa’s leading exponents of fish production. However, the finding from this study suggests a prospective lower antibiotic usage and possible threats of antimicrobials in the Egyptian and Nigerian fish production industry compared to poultry. Compared to fish research, the higher attention given to AMR research in poultry in South Africa may not necessarily represent a need to fill a knowledge gap in the sector. However, the poultry industry is the most important part of the South African agricultural economy. This importance may be responsible for the increasing trend of AMR studies in the sector compared to aquaculture.

Inappropriate antimicrobial use is a global issue with consequences for animals and humans. In this regard, some countries have established monitoring and control strategies to curb the corresponding menace. For example, In China and Brazil (two of the biggest producers of poultry meat globally), the increasing use of antibiotics in poultry for therapeutic and growth enhancement has led to increased selection pressures and a high prevalence of AMR in various bacterial species. As a result, regulations have been put in place due to the disturbing trend to reduce the overall administration of AMR and prohibit the use of particular antibiotics (Liu et al., 2016; Yang et al., 2017). Although some antimicrobials used as growth promoters are now prohibited in both countries (Walsh and Wu, 2016; Rabello et al., 2020); however, some are still used for therapeutic purposes (Gazal et al., 2021). In both cases, farms investigated reported using antimicrobials during animal production. As a result, a high AMR was observed, especially in ESBL strains, which also showed high AMR compared to other antimicrobials (Gazal et al., 2021). In India, there has been a shift in the national diet due to the younger urban population’s increased meat intake (Laxminarayan and Chaudhury, 2016). This shift has led to the intensive farming of food animals, resulting in the widespread use of antibiotics as growth promoters in these animals (Hosain et al., 2021). Although various cases of AMR have been observed across the countries mentioned above, regulatory framework and control policies seem to play a major role in regulating some antimicrobial use among farmers.

The challenge of AMR in food-producing animals is not just an African case but a global issue, especially in developing countries. For instance, the developing countries of South and Southeast Asia have established various intensive farming methods, thus, increasing animal population and antimicrobial consumption by food-producing animals (Coyne et al., 2019; Ferdous et al., 2019). Generally, because of increased meat consumption, antimicrobial use is anticipated to increase dramatically in some developing nations by 2030. According to Hosain et al. (2021), some of these countries include Vietnam (157%), Peru (160%), Indonesia (202%), Myanmar (205%), and Nigeria (163%). In Bangladesh (a developing country), approximately 94.16% of poultry farmers use antibiotics in their farms for growth enhancement and therapeutic purposes (Ferdous et al., 2019). The majority of antibiotics are made available directly to farmers as over-the-counter medication. Thus, over 60% of farmers use antibiotics outside the supervision of veterinarians (Masud et al., 2020). This implies that the tendency to develop AMR in such circumstances is high, especially relative to a lack of monitoring.

In aquaculture, some regions have regulatory limitations on the maximum residue levels allowed in edible components of animal-derived food items. In Europe, for example, food safety rules, including antimicrobial use in aquaculture, are quite stringent (Santos and Ramos, 2018). Furthermore, in the United States, the Food and Drug Administration (FDA), the government agency responsible for veterinary medicine, determines the criteria for antibiotic use, including acceptable modes of administration, dosage, withdrawal durations, and specificity for different species use (Santos and Ramos, 2018).

Most European countries allow the use of some antibiotics, such as sulfonamides, erythromycin, florfenicol, and oxytetracycline, in aquaculture (Lulijwa et al., 2020), and the United States allows the use of oxytetracycline, florfenicol, and sulfadimethoxine/ormetoprim (Love et al., 2020). This regulation (with proper monitoring strategies) enhances the regulation of inappropriate antimicrobial usage in the regions. In this regard, Norway can be seen as an example, as antimicrobial use regulation in salmon farming is highly stringent. Nevertheless, Norway reduced the usage of antimicrobials to negligible levels by improving diagnostics approaches and using more probiotics and vaccinations (Grave et al., 2006; Midtlyng et al., 2011). Furthermore, case studies in other European countries such as Denmark and Netherlands clearly illustrate the possibility of considerably reducing antimicrobial use while maintaining food quality and safety without negatively impacting the economy (Cogliani et al., 2011).

Despite strong regulation in some areas, the legal structure in some top aquaculture-producing countries is extremely limited. For example, India is the world’s second-largest aquaculture producer, accounting for 8% of total global production, yet antibiotic supplies and use are unregulated (Done et al., 2015; FAO, 2020). On the other hand, China is the largest exporter of fish and fisheries products and the largest aquaculture-producing nation, accounting for 67% of total global aquaculture (FAO, 2020). However, there are reported cases where animal antibiotics do not require veterinary prescriptions (Maron et al., 2013). Reports also indicate illegal antibiotics use, with residues in food animals and humans, implying a slack in regulation enforcement (Zhang et al., 2021).

### 4.3 Methods employed for antimicrobial susceptibility tests

Globally, different methods have been employed for antimicrobial susceptibility tests (AST), especially conventional phenotypic methods, which are the most commonly used. This study revealed that the methods used for AST were broth dilution, agar dilution, and disk diffusion. Dilution methods are based on microtitration plates, while diffusion methods are based on agar culturing (Ellner and Johnson, 1976; Horváth et al., 2016).

#### 4.3.1 Dilution Methods for antimicrobial resistance in fish and poultry research

One of the typical approaches to AST is the broth dilution test. This test determines the minimal inhibitory concentration (MICs) and involves diluting the antimicrobial agent in bacterial media. Specifically, a microdilution assay is the standard method to obtain MIC values (Marroki and Bousmaha-Marroki, 2021). However, researchers in this study rarely utilised dilution methods compared to diffusion methods.

The dilution methods include agar dilution, broth microdilution, and broth microdilution (Wiegand et al., 2008). Most reference laboratories use broth microdilution as the standard among the three methods. Broth microdilution involves twofold dilutions of antimicrobials made in a broth medium in a microtiter plate (Sykes and Rankin, 2013). The advantages of the dilution method are that it utilises minimal amounts of inoculum and has an associated low cost. It further allows for the assessment of various antibiotic drugs with individual isolates. However, it requires a lot of time, space, and reagents, and it also leaves room for human mistakes in preparing diluted antibiotics (Jorgensen and Ferraro, 1998).

Besides these traditional methods, several automated systems for AST, such as the Micro Scan WalkAway-96 System and the VITEK 2 System (bioMérieux), have been developed. These systems have been utilised for AST and identifying clinically relevant bacteria (Dade Behring). Examples include the E-test (developed by bioMérieux), which combines agar dilution and disk diffusion to deliver accurate and reliable quantitative results (Brown and Brown, 1991). Furthermore, unlike microdilution’s classification system, it further categorises resistant strains (Macias et al., 1994).

These traditional methods require more time than recently developed automated systems (Wiegand et al., 2008). On the other hand, automated systems reduce the labour required, provide rapid test results, allow for intra- and inter-laboratory standardisation, and possibly use artificial intelligence for data review (Winstanley and Courvalin, 2011). However, the system cards and panels are expensive and specifically formatted with antimicrobial agents and concentrations, which may be unsuitable for some species and experimental setups (Ferraro, 1994). These factors could play a role in the limited use of automated systems for AST observed in this study.

#### 4.3.2 Diffusion methods for antimicrobial resistance in fish and poultry research

The majority of the studies employed the disk diffusion method for AST. A disk diffusion method is a standardised approach to determining the infectious potential of rapidly dividing microorganisms (Clinical and Laboratory Standards Institute, 2015). Although introduced by Bauer and colleagues, the Clinical and Laboratory Standards Institute (CLSI) has continuously improved the method with specific procedures. Similar procedures are utilised by some global associations, such as the European Union Committee for Antimicrobial Susceptibility Testing (EUCAST), the British Society for Antimicrobial Chemotherapy (BSAC), and the American Society for Microbiology (ASM) (Baron et al., 2005; Andrews, 2008; EUCAST, 2022).

**Figure 7:**
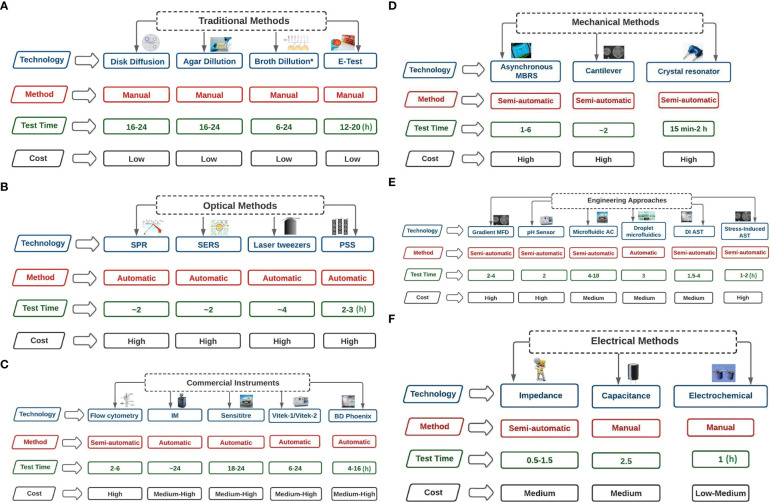
**(A)** Traditional Methods of AST technologies (test time in hours). Broth dilution* includes Broth microdilution. Broth dilution* includes Broth microdilution. Broth microdilution involves spreading antibiotics of varying concentrations over bacterial culture media. Agar dilution involves the spread of antibiotics of varying concentrations on agar plates, and bacteria are coated on the surfaces of the plates. In disk diffusion, filter paper with antibiotics in the desired quantities is applied on a surface of agar covered with microorganisms. The minimum inhibitory concentration (MIC) of antibiotics and antifungal drugs can be determined using the E-TEST, which consists of a preset gradient of antibiotic concentrations immobilised on a plastic strip. **(B)** Optical Methods of AST technologies (test time in hours). SPR=Surface plasmon resonance (refractive index is measured); PSS= Phase Shift Spectroscopy (optical analysis of Silicon micropillar topologies and suspended colonized bacteria); SERS = Surface-Enhanced Raman Scattering (involves using metallic nanoparticles to measure Raman scattered light signals); Laser tweezers involve Raman spectroscopy on the surface of an optically captured bacterial cell. **(C)** Commercial Instruments of AST technologies (test time in hours). IM= Isothermal Microcalorimetry (heat flowrate of bacterial suspension is measured in the absence or presence of antibiotics); Flow cytometry involves counting the viability of cells using dyes; In Sensititre, enzymatic activities of bacterial suspension are measured through fluorescence indicator in the presence of antibiotics; BD Phoenix uses the presence of antibiotics to record colorimetric changes and turbidity of bacterial suspension; Vitek 1 and 2 uses light attenuation from a photmeter to measure bacterial growth in the presence of antibiotics. **(D)** Mechanical Methods of AST technologies (test time in hours). Asynchronous Magnetic Bead Rotation Sensor (involves measuring the frequency of magnetic beads rotational); Crystal resonator measure of change in resonant frequency; Cantilever uses bacteria cells and measure fluctuations of the cantilever. **(E)** Engineering Approaches of AST technologies (test time in hours). DI AST= Dielectrophoresis induced Antibiotics Susceptibility Test (observes the behaviour of elongated bacteria during dielectrophoresis); Microfluidic agarose channel (microscope monitoring of bacteria immobilised in agarose); Gradient MFD = Gradient multifluidic (shows concentration gradient on the chip relative to different designs); Stress-Induced Antibiotics Susceptibility Test (microorganisms are immobilised in the presence and absence of antibiotics). **(F)** Electrical Methods of AST technologies (test time in hours). Electrochemical (measure changes in current relative to electrochemical reactions); Capacitance (measure capacitance of bacterial suspension); Impedance (measure impedance of bacterial suspension).

Disk diffusion offers several benefits; it is inexpensive, adaptable, and enables visibility of growth and the presence of mixed cultures and other abnormalities. Another advantage is that it can do direct susceptibility tests (DST) (Serisier et al., 2012). These advantages could be connected to the extensive use of this method for AMR studies across the study areas. However, EUCAST, BSAC, and the ASM are critical of DST because the inoculum is not usually standardised. A summary of methods used for AST is presented in **Figures 7A–F** (after Behera et al., 2019). Although several approaches are available for AST, methods such as electrical, engineering, and microfluidic were limited in this study.

### 4.4 Mechanism of action for antimicrobial resistance in fish and poultry research

An important contributor to the enormous challenges of AMR in humans is the poor management of antimicrobial drugs. Specifically, inappropriate prescription of antibiotic medication and an increased intake of antimicrobial drugs by animals and humans are two of the key contributors to the persistence of resistant microbes (Reygaert, 2018; Xavier et al., 2022).

Four main categories of AMR mechanisms are reported in the literature; active drug efflux (ADE), inactivation of drugs (ID), modification of drug targets (MDT), and limiting uptake of a drug (LUD). Among the four categories, limiting drug uptake was the most common mechanism of AMR reported in this study (both in fish and poultry). The result could be a consequence of the researcher’s interest in gram-negative bacteria compared to gram-positive bacteria. Both gram-positive and gram-negative bacteria exhibit AMR. However, the types of resistance mechanisms among these bacteria differ due to structural variability. Gram-negative bacteria use all four (LUD, MDT, ID, ADE) mechanisms. In contrast, gram-positive bacteria rarely use LUD due to the lack of a lipopolysaccharide outer membrane and lack of capacity for some types of drug efflux mechanisms (Reygaert, 2018).

Gram-negative bacteria can exhibit intrinsic resistance through drug efflux, drug inactivation, and limiting uptake. Intrinsic antibiotic resistance mechanisms are typically chromosome-encoded and include antibiotic-inactivating enzymes, permeability barrier mechanisms, and non-specific efflux pumps (Cox and Wright, 2013; Reygaert, 2018). These systems are hardwired into an organism’s fundamental genetic makeup and cannot be changed. Intrinsic antibiotic resistance mechanisms are usually found in bacteria (Cox and Wright, 2013), such as the antibiotic vancomycin resistance in E. coli. This resistance is caused by the permeability barrier of the outer membrane (Arthur and Courvalin, 1993).

On the other hand, acquired resistance mechanisms may be drug efflux, drug inactivation, and drug target modification and are typically attained through horizontal gene transfer (Cox and Wright, 2013; Reygaert, 2018). They include enzymes modifying the antibiotic or their target and plasmid-encoded specific efflux pumps (van Hoek et al., 2011). Modifying the antibiotic or its target poses a significant threat to animal and human health due to a shift from chromosomally-mediated resistance to plasmid-mediated resistance. Consequently, this has led to an increased rate of resistance expression and spread (Martinez, 2014).

#### 4.4.1 Antimicrobial resistance due to limiting uptake of drug

Limiting uptake of drugs is due to reduced transport and indicates that drugs become decreasingly effective with increasing use (Fernández et al., 2017). Studies have shown variations in the capacities of different bacteria types to restrict the intake of antimicrobial drugs. For studies on gram-negative bacteria (e.g., Ayandiran et al., 2018; Hafez et al., 2018; Mokgophi et al., 2021), the functions and shape of the lipopolysaccharide layer establish a barrier that prevents certain chemicals from entering the cell. As a result, certain bacteria developed an inherent resistance to particular classes of potent antimicrobial drugs (Blair et al., 2014). In addition, the amount of lipids in the outer membrane of bacteria could also influence drug uptake. For instance, the outer membrane of mycobacteria has a significant amount of lipids. Thus, studies with hydrophobic drugs (e.g., novobiocin, rifamycins, macrolides, aminoglycosides, etc.) observed easy penetration of the cell, while those with hydrophilic drugs observed restricted access (Lambert, 2002; Kumar and Schweizer, 2005).

Bacteria without a cell wall (e.g., Mycoplasma) are naturally resistant to all drugs that target the cell wall (Bébéar and Pereyre, 2005), including beta-lactams and glycopeptides. The absence of an outer membrane in gram-positive bacteria thus eliminates the issue of drug inaccessibility to these bacteria. The inherent resistance of enterococci to aminoglycosides (e.g., Ngbede et al., 2017; Molechan et al., 2019) is connected to its cell wall, which gives polar molecules a hard time for penetration. As a result, the bacteria become resistant to antimicrobial drugs, such as *E. faecalis*, *E. faecium*, *E. durans*, *E. mundtii*, *E. raffinosus*, and *E. gallinarum* observed in this study.

#### 4.4.2 Antimicrobial resistance due to modification of drug targets

This study observed limited studies that reported modification of drug targets as a mechanism of AMR. However, Adelowo et al. (2014) reported this mechanism of AMR in a study on *Escherichia coli* resistance to tetracycline, gentamicin, and neomycin. There are many different parts of the bacterial cell that have the potential to be targets of antimicrobial medications. There are also many different parts of the cell that the bacteria could change to develop drug resistance. One of the ways that bacteria can develop resistance to -lactam antibiotics is by modifying the penicillin-binding proteins (PBPs) structure and/or number. PBPs are transpeptidases, majorly involved in peptidoglycan formation in the cell wall. Numerical changes in PBPs affect the binding ability of drugs and the amount of drug that can bind to that target (Reygaert, 2018). For example, a modification to the PBPs structure can either prevent drugs from binding to the protein or lessen its binding capacity, such as methicillin resistance (conferred by mecA gene expression, which encodes PBP2 in S. aureus (Blair et al., 2015).

Other examples include the development of resistance to the antibiotic vancomycin, which has become a significant problem in both *Staphylococcus aureus* and enterococci. The acquisition of van genes is the mechanism that underlies the development of resistance to vancomycin (Beceiro et al., 2013). This mechanism leads to the structural modification of peptidoglycan precursors, resulting in reduced binding capacity to its targets. Furthermore, antibiotic resistance to the ribosomal subunits can occur through ribosomal protection, ribosomal subunit methylation, and ribosomal mutation. These mechanisms prevent drug attachment to the ribosome (Eliopoulos et al., 2003; Kumar et al., 2013). Finally, for antibiotics that block metabolic pathways, resistance develops through the overproduction of resistant dihydropteroate synthase, dihydrofolate reductase enzymes (trimethoprim), and dihydropteroate synthase (sulfonamides) (Reygaert, 2018).

#### 4.4.3 Antimicrobial resistance due to inactivation of drugs

Our study observed limited publications that reported drugs as a mechanism of AMR in their studies. Bacteria primarily inactivate drugs either through drug breakdown or by chemical group transfer to the drug. Beta-lactamases are a pervasive family of enzymes involved in drug hydrolysis, e.g., tetracycline through the tetX gene (Kumar et al., 2013; Blair et al., 2015).

#### 4.4.4 Antimicrobial resistance due to active drug efflux

The primary purpose of efflux pumps is to clear the bacterial cell of potentially harmful molecules (Soto, 2013). Bacteria have genes for efflux pumps that are chromosomally encoded in their DNA. Others are induced or overexpressed on the availability of suitable substrate or in response to specific environmental stimuli. Some genes express themselves constantly, while others only become active in response to external factors. Depending on the type of carbon source that is accessible, the resistance capacity of these pumps can be affected (Villagra et al., 2012; Blair et al., 2014).

**Figure 8:**
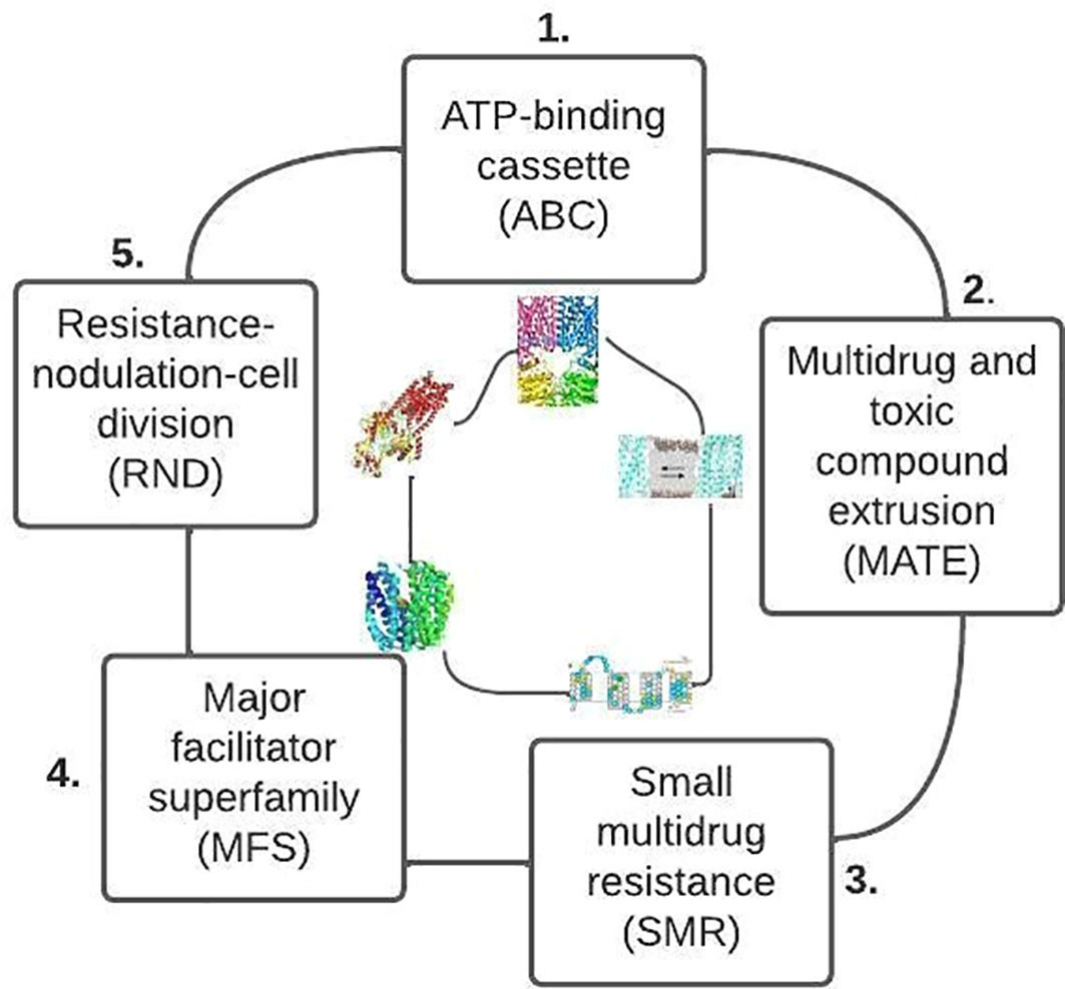
Primary families of bacteria efflux pump based on their structure and energy source (after Reygaert, 2018).

Most bacteria have diverse efflux pumps across the primary families (**Figure 8**). In gram-positive bacteria (e.g., fluoroquinolone efflux pumps), efflux pumps can bestow an inherent resistance because they are encoded on the chromosome. On the other hand, the efflux pumps are extensively dispersed in gram-negative bacteria and can originate from any of the five families. However, the efflux pumps that belong to the Resistance-nodulation-division (RND) family transporters are the most clinically significant (Blair et al., 2014; Kourtesi et al., 2013).

### 4.5 Environment and public health implications of antimicrobial residues

In this study, the various antimicrobial resistance genes (ARGs) reported (section 3.3) indicate the spread of modifications in antimicrobial drug targets. Thus, animal or human exposure to these genes poses a significant risk. There is widespread agreement that ARGs in sediments pose a possible risk to the surrounding environment (Wang et al., 2015). ARGs may accumulate and be transmitted through the sediments (Marti et al., 2014) to aquatic organisms and humans. Studies have reported many ARGs in the water and sediment of aquaculture farms with the ability to encode resistance to different classes of antibiotics (Shen et al., 2020). Evidence supports the hypothesis that, either directly or indirectly, nutrients have a role in the spread of ARGs (Zhang et al., 2022). Thus, consistent and prolonged addition of phosphorus and nitrogen alters the bacterial community makeup and contributes to the environmental spread of ARGs (Pan et al., 2020).

Antimicrobial residues have diverse adverse effects on biota at various environmental trophic levels. They have the potential to be ecotoxic to non-target aquatic and terrestrial organisms (Chee-Sanford et al., 2012; Jechalke et al., 2014). In the terrestrial environment, the residues can enter the plant’s body and obstruct physiological functions like germination, photosynthesis, and growth (Minden et al., 2017). Once these ARGs are introduced into the human symbiotic bacteria, they pose significant threats to the ecological environment and human health (Forsberg et al., 2012; Zhang et al., 2022). For instance, there has been a significant increase in the number of antibiotic-resistant bacteria linked to food-borne diseases (Caniça et al., 2019). A few studies have indicated a direct relationship between antibiotic use in food animals and the evolution of AMR in human pathogens (Teuber, 1999; Van den Bogaard et al., 2002), making therapy ineffective.

Besides, human pathogens (e.g., *Vibrio* spp., *Shigella* spp., *Salmonella* spp.) and opportunistic pathogens (e.g., *Edwardsiella tarda*, *Aeromonas hydrophila*) may directly transmit AMR to humans from aquatic environments. Antimicrobial residues may be transmitted by eating or handling seafood, drinking water, or direct contact with water or aquatic species. Upon entry into the human biological system, they may cause allergic responses, cancer, or changes in the native gut microflora composition. Other negative implications on human health include increased infection severity and a higher rate of treatment failures (Darwish et al., 2013).

### 4.6 Government policy against antimicrobial resistance

Recent projections indicate that AMR has already impacted global sustainable development and requires multisectoral engagement as a response (Hoffman et al., 2015). Over the years, various government policies have been established to limit antimicrobial overuse and misuse. For instance, antimicrobial use as a growth promoter has been banned since 2016 in all European Union member states (Serwecińska, 2020). Various public awareness campaigns, guidelines on antimicrobial usage, and best practices to ensure safe antimicrobial use have been reported. Examples include; (i) antimicrobial use with strict adherence to the prescription of a qualified and certified doctor, (ii) complete administration of antimicrobials prescribed for use to avoid the risk of re-infection and development of AMR, (iii) administration of drugs only to the specified animal, specified age, specified breed, etc. (Serwecińska, 2020).

Further, the World Health Organization (WHO) has developed a Global Action Plan (GAP) with a One Health approach to address AMR (WHO, 2015). Other strategies developed based on the GAP include optimising antimicrobial use, reducing the incidence of infection, and improving awareness and understanding of AMR (Serwecińska, 2020). Furthermore, besides public awareness campaigns and guidelines, political actions on AMR have been established, such as the Berlin Declaration of the G20 Health Ministers (G20 Health Ministers, 2017) and the 2016 United Nations resolution (United Nations General Assembly, 2016). These are all pointers to global actions and interventions against AMR.

However, there are challenges faced in addressing AMR. Most available policy options on antimicrobial use have not been rigorously evaluated. Moreover, some countries are yet to implement government policies to reduce antimicrobial use (WHO, 2018). In addition, there are poor controls and oversight on the availability of antimicrobials in many countries and unnecessary pressures on prescribers of antimicrobials from animal owners (FAO/OIE/WHO, 2004; O'Neill, 2016).

To adequately address AMR on a global scale, all countries should develop an institutional framework to oversee, regulate, and enforce compliance with international guidelines. There is also a need to evaluate available intervention strategies for AMR. Evaluating available intervention strategies will allow for effective planning and mapping out future directions for public use against AMR.

### 4.7 Future research directions on antimicrobial resistance: Considerations for One Health

One Health embraces environmental considerations as well as plant, animal, and optimal human health (Zinsstag et al., 2012; Finley et al., 2013). Hence, future research needs to incorporate One Health measures to address AMR in the wider environment since most of them have genes that confer resistance to environmental origins (Collignon, 2012; Gaze et al., 2013; Holmes et al., 2016). In addition, future research should also incorporate environmental risk assessment and monitoring. This incorporation will allow for identifying more specific measures to address AMR in the environment. It will also offer a clearer contextual perspective and a better understanding of the environmental roles in AMR selection and spread (Walsh et al., 2011; Ashbolt et al., 2013; Huijbers et al., 2015).

There is a need to further investigate the presence of antibiotics, especially in chicken after it has been cooked. This research question needs to be answered because the antibiotic risk to human health is likely minimal in some circumstances and high in others. However, adequate cooking eliminates germs in food but does not destroy some antibiotics whose melting point exceeds the cooking temperature.

There is also a need to establish how long different antibiotics remain effective across different fish and poultry species (especially chickens). After administration, antibiotics begin to work immediately, and the biological half-life of each antibiotic may vary. Such research will allow farmers to establish their metabolic time and, thus, prevent antibiotic residues from entering the food chain.

### 4.8 Conclusions

The number of publications on antimicrobial resistance research in fish and poultry varies among the three countries, with more focus on poultry. Egypt leads AMR research with more publications in the research field in recent years than South Africa and Nigeria. In Nigeria, the number of publications increased from 2010 to 2021. This period was when most antimicrobial resistance research was conducted. The number of research publications in South Africa increased from 1992, with greater output from 2017 upwards. However, the finding from this study might not reflect the level of prevalence or issues relating to AMR across the study areas. Instead, it could be influenced by various factors such as funding, availability of relevant and skilled human resources, research interest, etc.

Furthermore, the disk diffusion method is majorly used for antimicrobial susceptibility tests in fish and poultry among researchers in the countries under study. Disk diffusion offers several benefits; it is inexpensive, adaptable, and enables visibility of growth and the presence of mixed cultures and other abnormalities. These advantages could be connected to the extensive use of this method for AMR studies in the countries under study. Limiting drug uptake is the most commonly reported mechanism of AMR in this study (both in fish and poultry). This mechanism is due to reduced transport and indicates that drugs become decreasingly effective with increasing use.

Generally, this study reveals public and environmental health threats and suggests the need to urgently promote and advance AMR research, particularly for countries on the global hotspot for antimicrobial use. Furthermore, regarding the effects of AMR on humans and animals, it is necessary to intensify research, policy, and practical efforts to promote sustainable antimicrobial use.

## Data availability statement

The original contributions presented in the study are included in the article/**Supplementary material**. Further inquiries can be directed to the corresponding author.

## Author contributions

EO: Conceptualisation, Methodology, Supervision, Validation, Formal analysis, Writing- Original draft preparation. RO Validation, Writing- Reviewing and Editing. BA: Validation, Writing- Original draft preparation. JE: Data curation, Writing- Original draft preparation. OA: Data curation, Investigation, Validation. AB: Data curation, Validation. NM: Writing- Reviewing and Editing. : BF: Formal analysis, Writing- Original draft preparation. AT: Writing- Original draft preparation. IO: Data curation, Investigation. OA: Writing- Reviewing and Editing. All authors contributed to the article and approved the submitted version.

## Conflict of interest

The authors declare that the research was conducted in the absence of any commercial or financial relationships that could be construed as a potential conflict of interest.

## Publisher’s note

All claims expressed in this article are solely those of the authors and do not necessarily represent those of their affiliated organizations, or those of the publisher, the editors and the reviewers. Any product that may be evaluated in this article, or claim that may be made by its manufacturer, is not guaranteed or endorsed by the publisher.
