# Supplementary material for: Antimicrobial resistance in fish and poultry: Public health implications for animal source food production in Nigeria, Egypt, and South Africa
Source: Front Antibiot. 2022 Nov 10;1:1043302. doi: 10.3389/frabi.2022.1043302 (PMC11732016; doi:10.3389/frabi.2022.1043302)
Supplement: Supplementary file 1 [file DataSheet_1.docx]

**Table 2**. Characteristics of selected publications on antimicrobial resistance in fish and poultry in South Africa.

| **Antimicrobial Resistance** | **Pathogen** | **Method** | **Sample** | **Mechanism of Resistance** | **References** |
| --- | --- | --- | --- | --- | --- |
| **Poultry** |  |  |  |  |  |
| 6.7% of *Escherichia coli* tested were multidrug-resistant, and 67.3% were resistant to at least one antibiotic. | *Escherichia coli* | Disc diffusion | Swab Carcasses  Ceacal | Limiting uptake | Mclver et al., 2020 |
| Resistance of *E. faecium* to quinupristin-dalfopristin was found in 24% of the sample. Across all species, there were twenty-four antibiograms for multidrug resistance. | *Enterococcus* spp. | Disc diffusion | Swab Carcasses  Ceacal | Limiting uptake | Molechan et al., 2019 |
| All of the antimicrobial drugs employed on *Salmonella* isolates from chickens were ineffective against the pathogen. | *Salmonella* isolates | Disc diffusion | Ceacal | Limiting uptake | Zishiri et al., 2016 |
| Tetracycline-resistant isolates (98.9 to 100%) were found in commercially raised birds. Gentamicin resistance was 1.6%, while streptomycin resistance was 11.5% of the population. | *Campylobacter* bacteria | Agar dilution | Caeca | Limiting uptake | Bester and Essack, 2012 |
| Commercially reared birds had a high level of tetracycline resistance (98.9 to 100%). Streptomycin and gentamicin resistance was found in 11.5% and 1.6% of the population, respectively. | *Campylobacter jejuni* | Agar dilution | Caeca | Limiting uptake | Bester and Essack, 2008 |
| 96.6% of *Salmonella* isolates had multidrug resistance profiles. In addition, more than nine antibiotic resistance was detected in isolates. | *Salmonella* isolates | Disc diffusion | Swab | Limiting uptake | Akinola et al., 2019 |
| 71.4% of *Escherichia coli* isolates were resistant to at least one antimicrobial agent. | *Salmonella* spp., *Escherichia coli* | Disc diffusion | Table eggs | Limiting uptake | Adesiyun et al., 2020 |
| Most *Escherichia coli* isolates from broilers had minimum inhibitory concentration (MIC) values that indicated resistance to enrofloxacin–75.6%, nalidixic acid-90.5%, fosfomycin, and doxycycline-98.2% each, sulphamethoxazole-78.7%, etc. | *Escherichia coli* | Agar dilution | Carcasses | Limiting uptake | Oguttu et al., 2008 |
| 81.8% of the *Salmonella* samples were multidrug-resistant. | Salmonella | Disc diffusion | Killed, swabs | Limiting uptake | Mokgophi et al., 2021 |
| Oxytetracycline – 10%, ampicillin - 16.7%, and trimethoprim - 36.7% were *Salmonella* isolates' most resistant antimicrobial agents. | Salmonella | Broth dilution | Rectal and cloacal swabs | Limiting uptake | Mathole et al., 2017 |
| *S. aureus* had a multiple antibiotic resistance index of 0.23%, and 39.17% of the sample were multidrug-resistant. | *Staphylococcus aureus* | Disc diffusion | Faecal, swab, litter, killed | Limiting uptake | Amoako et al., 2020 |
| 21% of *S. aureus* were methicillin-resistant. In addition, multidrug resistance was found in 22.2% of the isolates. | *Staphylococcus aureus* | Disc diffusion | Meat products | Limiting uptake | Govender et al., 2019 |
| A variety of antibiotic classes can be resistant to *Campylobacter* spp. | *Campylobacter* spp. | Broth dilution | Faecal | - | Reddy and Zishiri, 2017 |
| Antibiotic resistance to three or more antibiotics (trimethoprim, tetracycline, nalidixic acid, ciprofloxacin, ampicillin, and gentamicin) was found in 40% of the isolates. | *K. ozaenae, K. rhinoscleromatis* | Disc diffusion | Neck-skin | Limiting uptake | Fielding et al., 2012 |
| All of the methicillin-resistant staphylococcus isolates tested positive for multiple-drug resistance. | *Staphylococcus aureus* | Disc diffusion | Poultry farm, abattoir | - | Amoako et al., 2019 |
| Resistance to antibiotics at the phenotypic level was extremely high. | *Staphylococcus aureus* | Disc diffusion | Faecal | Limiting uptake | Adegoke and Okoh, 2014 |
| **Fish** |  |  |  |  |  |
| *Aeromonas spp*. showed high level of resistance to augmentin - 86.5%, amoxicillin - 89.2%, and tetracycline - 78.3%. | *Aeromonas* spp. | Disc diffusion | Swabs | Limiting uptake | Jacobs and Chenia, 2007 |
| Antibiotic resistance to clindamycin, rifampicin, ampicillin and erythromycin ranged from 67 to 81%. | *Staphylococcus aureus* | - | Gill and skin tissues | - | Fri et al., 2020 |
| A high prevalence (62.9%) of *S. aureus* with the presence of Methicillin-resistant *S. aureus* strains (16.3%). | *Staphylococcus aureus* | Disc diffusion | Gill and skin tissues | Limiting uptake | Fri et al., 2018 |

**Table 3**. Characteristics of selected publications on antimicrobial resistance in fish and poultry in Egypt.

| **Antimicrobial Resistance** | **Pathogen** | **Method** | **Sample** | **Mechanism of Resistance** | **Reference** |
| --- | --- | --- | --- | --- | --- |
| **Poultry** |  |  |  |  |  |
| Tetracycline residues in poultry litter pose severe environmental concerns, as measured by a hazard quotient greater than 1. (1.64) | *Escherichia coli*, *Salmonella* spp. | Disc diffusion | Litter, faecal, and water | - | Dahshan et al., 2015 |
| 100% of *Escherichia coli* isolates showed resistance to sulfamethoxazole-trimethoprim, ampicillin, and erythromycin. | *Escherichia coli* | Disc diffusion | Organs | Limiting uptake | Ibrahim et al., 2019 |
| A 66.3% incidence of multidrug-resistant of *Escherichia coli*. (Significantly higher in the fresh sample than frozen and chilled). | *Escherichia coli* | Disc diffusion | Killed | Limiting uptake | Abdelkarim et al., 2020 |
| Molecular identification of the antibiotic resistance gene indicated a 100% blatem in all *Escherichia coli* and salmonellae samples | *Escherichia coli* and salmonellae | - | Organs | - | Kamel et al., 2021 |
| *Escherichia coli* showed resistance to cefotaxime (33.3%), streptomycin (61.9%), ampicillin (71.4%), and tetracycline (80.9%). | *Salmonella enterica Escherichia coli* | Disc diffusion | Killed, Organs | - | Moawad et al., 2017 |
| 87.5% of *P. aeruginosa* were resistant to florfenicol, 86.9% of *Salmonella* serovars demonstrated colistin sulphate resistance, 48% of *Escherichia coli* were resistant to norfloxacin. | *Salmonella spp*., *E. coli*, and *P. aeruginosa* | Disc diffusion | Organs | Limiting uptake | Iraqi et al., 2021 |
| All tested isolates indicated the presence of tetracyclines and β-lactams resistance genes. | *Escherichia coli* | Disc diffusion | Organs | Limiting uptake | Ali et al., 2019 |
| In avian pathogenic *Escherichia coli*, qacs resistance genes and virulence genes (papc, iss,) are common. | *Escherichia coli* | Disc diffusion | Organs | Limiting uptake | Enany et al., 2019 |
| The establishment of colistin resistance and multidrug resistance in Enterobacteriaceae | *E. coli*, *E. cloacae*, *K. pneumoniae* and *Citrobacter spp*. | Broth dilution | Swaps | Limiting uptake | Moawad et al., 2018 |
| The most resistant antibiotics were chloramphenicol, nalidixic acid, tetracycline, and ampicillin. | *Escherichia coli* | Disc diffusion | Organs | Limiting uptake | Awad et al., 2016 |
| More than two of the ten antimicrobial drugs evaluated were resistant to salmonella serovars. | *Salmonella* serovars | Disc diffusion | Organs | Limiting uptake | Mahmoud et al., 2018 |
| Over 80% of afec and 70% of apec were multidrug resistant | *Escherichia coli* | - | Killed, swabs, litter | - | Hussein et al., 2013 |
| None of the isolates exhibited imipenem resistance. | *Salmonella enterica* | Disc diffusion | Killed and Swabs | Limiting uptake | Elkenany et al., 2019 |
| A low resistance (57.7%) to cefuroxime and high resistance (100%) of *Salmonella* spp. to trimethoprim/sulfamethoxazole. | *Salmonella* spp | Disc diffusion |  | - | Elkenany et al., 2018 |
| All isolates of *S. enterica* were sensitive to all antimicrobials tested. | *Salmonella enterica* | Disc diffusion | Organs | - | El-sharkawy et al., 2017 |
| *Salmonella* isolates showed multidrug resistance (75.9%), extensively-drug resistance (16.5%), and pan-drug resistance (5.1%). | *Salmonella spp* | Disc diffusion | Killed | - | Elshebrawy et al., 2021 |
| Antimicrobial resistance genes were widely distributed among *Salmonella* isolates. | *Salmonella* isolates | Disc diffusion | Organs | Limiting uptake | Ammar et al., 2019 |
| A high prevalence of multidrug-resistant *Salmonella heidelberg* was observed in chickens. | *Salmonella* isolates | Disc diffusion | Swabs | - | Elhariri et al., 2020 |
| Amoxicillin-clavulanic acid (63%), erythromycin (67.2%), and rifamycin (82.7%) exhibited substantial levels of resistance. | *Enterobacteriaceae* isolates | Disc diffusion | Organs | - | Ei-demerdash et al., 2018 |
| 100% of *Helicobacter pylori* were resistant to streptomycin; 42.9% to norfloxacin, chloramphenicol, and neomycin; 57.1% to erythromycin and sulfamethoxazole; 71.4% to ampicillin, oxytetracycline, and nalidixic acid; and 85.7% to penicillin and amoxicillin. | *Helicobacter pylori* | Disc diffusion | Killed and Organs | - | Hamada et al., 2018 |
| Highest resistance of *Salmonella* isolates (100% each) to tetracycline and erythromycin and the least (16.7%) resistance to ceftriaxone. | *Salmonella* spp | Disc diffusion | Product | Limiting uptake | Gharieb et al., 2015 |
| Highest resistance of *Salmonella* isolates (100% each) to amoxicillin, penicillin, and erythromycin. Ampicillin (91.6%), oxytetracycline (95.2), sulphamethoxazole (96.4%), and nalidixic acid (98.8%) also indicated high resistance of *Salmonella* isolates. | *Salmonella* isolates | Disc diffusion | Killed | Limiting uptake | Abd-elghany et al., 2015 |
| 5.1%, 16.5%, and 75.9% of the isolates identified were classified as pan-drug resistant, extensively-drug resistant, and multidrug-resistant, respectively | *Salmonella* isolates | Broth microdilution, Disc diffusion | Killed | Limiting uptake | Elshebrawy et al., 2021 |
| **Fish** |  |  |  |  |  |
| 40% of the samples showed resistance to cefotaxime, 56% were resistant to ceftazidime, 84% to ampicillin, and 100% to cefoxitin. | *Aeromonas hydrophila* | Disc diffusion | Killed | - | Ramadan et al., 2018 |
| *Aeromonas hydrophila* isolated from fish exhibited a variety of drug resistance and virulence factors. | *Aeromonas hydrophila* | Disc diffusion | Swabs | - | Ahmed et al., 2018 |
| All isolates were resistant to at least three of the antimicrobials tested. | *Aeromonas spp* | Disc diffusion | Swabs | - | Zahran et al., 2019 |
| *Aeromonas hydrophila* isolates showed resistance to sulphamethoxazole and cefotaxime (80% each), streptomycin, erythromycin, and cloxacillin (100% each). | *Aeromonas spp* | Disc diffusion | Organ | - | Hafez et al., 2018 |
| Recovered strains showed high resistance to amoxicillin and complete sensitivity to ciprofloxacin. | *Aeromonas hydrophila* | Disc diffusion | Organ | Limiting uptake | Algammal et al., 2020 |
| 90.9% isolates of *Aeromonas* *hydrophila* showed sensitivity to doxycycline and florfenicol. 68.18% showed susceptibility to ciprofloxacin and oxytetracycline. | *Aeromonas* and *Pseudomonas* spp. | Disc diffusion | Organs | Limiting uptake | El-bahar et al., 2019 |
| *Enterobacteriaceae* isolates were found to be resistant to carbapenems and cephalosporins in the majority of cases. | *Enterobacteriaceae* isolates | Disc diffusion | Organ | - | Hamza et al., 2020 |
| 33.2% of isolates exhibited multidrug resistance phenotypes and had at least one gene for antimicrobial resistance. | Gram-negative bacteria | Disc diffusion | Water | - | Ishida et al., 2010 |

**Table 4**. Characteristics of selected publications on antimicrobial resistance in fish and poultry in Nigeria.

| **Antimicrobial Resistance** | **Pathogen** | **Method** | **Sample** | **Mechanism of Resistance** | **Reference** |
| --- | --- | --- | --- | --- | --- |
| **Poultry** |  |  |  |  |  |
| To varying degrees, all of the isolated strains developed resistance to antibiotics. | *Escherichia coli* | Disc diffusion | Faecal | Limiting uptake | Oluduro, 2012 |
| The antibiotic resistance patterns of *Salmonella* and *E. coli* isolates displayed strikingly different levels of variation. | *Salmonella* spp and *Escherichia coli* | Disc diffusion | Swap | - | Nabawy et al., 2016 |
| Presence of *Escherichia coli* strains capable of producing extended-spectrum beta-lactamase (esbl) in birds from commercial poultry farms operating on a small scale. | *Escherichia coli* | Disc diffusion | Swap | - | Kwoji et al., 2019 |
| *Escherichia coli* bacteria isolated from poultry droppings are more resistant to antibiotics than those isolated from cow manure. Resistance to all antibiotics except meropenem is prevalent. | *Escherichia coli* | Disc diffusion | Swap and Faecal | Limiting uptake | Egbule and Yusuf, 2019 |
| All esbl- and ampc-positive bacteria were highly resistant to the antibiotics tested, particularly cephalosporins and carbapenems. | *Escherichia coli*, *Klebsiella* spp. and *Pseudomonas* spp. | Disc diffusion | - | - | Nnaji et al., 2021 |
| Prevalence of apec isolates in rural chickens that were resistant to various antibiotics. | *Escherichia coli* | Disc diffusion | Swap | Limiting uptake | Geidam et al., 2012 |
| 61% of *Escherichia coli* strains showed reduced susceptibility to ciprofloxacin | *Escherichia coli* | Disc diffusion | Killed | - | Fortini et al., 2011 |
| Gene for tetracycline resistance was detected in the investigated poultry farms. | Enterococcal strains | Disc diffusion | Faecal | - | Ayeni et al., 2016 |
| *Escherichia coli* isolates were multi-drug-resistant to ciprofloxacin, chloramphenicol, gentamycin, ampicillin, streptomycin, erythromycin, tetracycline, and enrofloxacin. | *Escherichia coli* | Disc diffusion | Killed and Swap | Limiting uptake | Nwankwo et al., 2021 |
| Day-old commercial broiler chicks were colonised with multidrug-resistant coliforms Klebsiella and *Escherichia coli.* | *Klebsiella* spp, *Escherichia coli,* | Disc diffusion | Swap | Limiting uptake | Okorafor et al., 2019 |
| 81% of *Escherichia coli* strains showed resistance to tetracycline (among the highest), gentamicin - 8%, and neomycin - 14% (among the lowest). | *Escherichia coli* | - | Waste, litter, soil, and water | Modifying drug target | Adelowo et al., 2014 |
| *Escherichia coli* and *Staphylococcus aureus* showed high resistance to penicillin, lincomycin, tylosin, tetracycline, chloramphenicol, and ampicillin with mic values >8.0 g/l. | *Escherichia coli* and *Staphylococcus aureus* | Micro-broth dilution | Killed | - | Mamza et al., 2010 |
| Tetracycline resistance was shown by 95% of the esbl/pampc-producing strains. Others were gentamicin (55%), chloramphenicol (60%), and trimethoprim/sulfamethoxazole (80%). | *Escherichia coli*, *K. pneumonia*, *E. asburiae,* and *Providencia* spp. | Disc diffusion | Killed | - | Chah et al., 2018 |
| *Salmonella* isolates showed a 100% resistance to ampicillin, doxycycline, and tetracycline. Resistance to neomycin, ceftazidime, nalidixic acid, and sulphamethoxazole were 28.6%, 78.6%, 85.8%, and 92.9%, respectively. In addition, all the isolates showed susceptibility to ciprofloxacin. | *Salmonella* isolates | Disc diffusion | Live | Limiting uptake | Omoshaba et al., 2017 |
| All isolates were resistant to tetracycline, streptomycin, neomycin, enrofloxacin, norfloxacin, ciprofloxacin, chloramphenicol, and ampicillin. | *Klebsiella* spp, *Escherichia coli*, *E. aerogenes,* and *Salmonella* spp. | Micro-broth dilution | Swab | Limiting uptake | Ojo et al., 2012 |
| Multidrug resistance was exhibited in 64.3% of *Staphylococcus aureus* isolates from chicken carcasses. | *Staphylococcus aureus* | - | Swab | Limiting uptake | Okorie-Kanu et al., 2020 |
| The AST results revealed the highest resistance of 55 and 91% to carbenicillin and ceftazidime, respectively. | *Pseudomonas aeruginosa* | - | Faecal | - | Odumosu et al., 2016 |
| Species identified with high resistance to trimethoprim, tetracycline, and quinolones. | *E. coli* | Disc diffusion | Faecal | Limiting uptake | Ayandiran et al., 2018 |
| Antimicrobial resistance genes detected. 7% of *Salmonella enterica* isolates were multidrug-resistant. | *Salmonella enterica* | Disc diffusion | Faecal | - | Ajayi et al., 2019 |
| The most common phenotypic resistance was to sulphonamides (71.6%), nalidixic acid (79.7%), gentamicin (48.6%), and ciprofloxacin (50.0%). | *Salmonella* isolates | - | Faecal, litter | - | Jibril et al., 2021 |
| Different resistance patterns were observed, and 53.1% of the isolates indicated multidrug resistance. | *Enterococcus* spp. | Disc diffusion | Faecal, swab | Limiting uptake | Ngbede et al., 2017 |
| All isolates tested positive for antimicrobial resistance, with MIC ranging from 16mug/ml to 512mu g/ml. | Gram-negative bacteria | Broth dilution | Water | - | Adelowo and Fagade, 2012 |
| *Escherichia coli* isolates showed resistance to gentamicin, ciprofloxacin, cephalothin, ampicillin, and sulfamethoxazole at 13.6%, 18.2%, 13.6%, 22.7%, and 54.5% respectively. Overall, 65.4% of the isolates showed resistance to multiple antibiotics. | Proteus, Staphylococcus, and *Escherichia coli* | Disc diffusion | Faecal | Limiting uptake | Olonitola et al., 2015 |
| *Escherichia coli* showed resistance to ampicillin (34.1%), trimethoprim/sulfamethoxazole (39.8%), and tetracycline (58.8%). | *Escherichia coli* | Broth microdilution | Faecal | Limiting uptake | Adenipekun et al., 2015 |
| **Fish** |  |  |  |  |  |
| Drug resistance of isolates ranged from 50% to 90%., with bacillus species having the highest resistance. | *Bacillus* species | Disc diffusion | Killed | Limiting uptake | Ayandiran and Dahunsi, 2017 |
| 67.6% of Vibrio isolates were multiple drug-resistant. | *Vibrio* isolates | Disc diffusion | Water | Limiting uptake | Igbinosa, 2016 |
| All isolated *Escherichia coli* were resistant to at least one antibiotic. | *Escherichia coli* | Disc diffusion | Water, sediment | Limiting uptake | Ajewole et al., 2021 |
| High-level resistance to cefotaxime (42.9%), trimethoprim (85.7%), and tetracycline (96.1%) | *Escherichia coli* | Disc diffusion | Organ | - | Odumosu et al., 2021 |
